# Tunability Limit of Photoluminescence in Colloidal Silicon Nanocrystals

**DOI:** 10.1038/srep12469

**Published:** 2015-07-22

**Authors:** Xiaoming Wen, Pengfei Zhang, Trevor A. Smith, Rebecca J. Anthony, Uwe R. Kortshagen, Pyng Yu, Yu Feng, Santosh Shrestha, Gavin Coniber, Shujuan Huang

**Affiliations:** 1 Australian Centre for Advanced Photovoltaics, University of New South Wales, Sydney 2052, Australia; 2 School of Chemistry, University of Melbourne, Parkville, VIC, 3010 Australia; 3 Department of Mechanical Engineering, University of Minnesota, Minneapolis, Minnesota 55455, United States; 4 Research Center for Applied Sciences, Academia Sinica, Taipei, Taiwan

## Abstract

Luminescent silicon nanocrystals (Si NCs) have attracted tremendous research interest. Their size dependent photoluminescence (PL) shows great promise in various optoelectronic and biomedical applications and devices. However, it remains unclear why the exciton emission is limited to energy below 2.1 eV, no matter how small the nanocrystal is. Here we interpret a nanosecond transient yellow emission band at 590 nm (2.1 eV) as a critical limit of the wavelength tunability in colloidal silicon nanocrystals. In the “large size” regime (d > ~3 nm), quantum confinement dominantly determines the PL wavelength and thus the PL peak blue shifts upon decreasing the Si NC size. In the “small size” regime (d < ~2 nm) the effect of the yellow band overwhelms the effect of quantum confinement with distinctly increased nonradiative trapping. As a consequence, the photoluminescence peak does not exhibit any additional blue shift and the quantum yield drops abruptly with further decreasing the size of the Si NCs. This finding confirms that the PL originating from the quantum confined core states can only exist in the red/near infrared with energy below 2.1 eV; while the blue/green PL originates from surface related states and exhibits nanosecond transition.

Colloidal silicon nanocrystals (Si NCs) have attracted tremendous research interest due to their intriguing optical and electronic properties and thus promising applications from microelectronics to optoelectronics compatible with silicon-based optoelectronic integrated circuits^1^,^2^,^3^,^4^,^5^. One of the most important features of NCs/quantum dots (QDs) is the broad tunability of their photoluminescence (PL) energy induced through quantum confinement by changing the nanocrystal size^3^,^6^. The PL of Si NCs has been extensively investigated^7^,^8^,^9^. Two PL bands are prominent; one in the red/near infrared (referred to as S-band with μs lifetime) and one in blue/green PL (referred to as F-band with ns lifetime). PL quantum yield (QY) as high as 60% have been reported for colloidal ensemble of Si NCs^10^,^11^,^12^. It is generally accepted the PL in the red/NIR dominantly originates from the quantum confinement while the green/blue PL is arisen from the oxygen relevant defect/surface states^1^,^4^,^5^,^13^,^14^,^15^,^16^,^17^. The exciton emission exhibits a marked blue shift with decreasing the size which can be predicted based on quantum confinement in the large size regime^18^. Great effort has been made for broadening the tunability of the PL by changing the size of Si NCs^19^,^20^. However, it has been confirmed that the PL peak does not further blue-shift when the size decreases smaller than 2 nm or the PL peak approaches 590 nm in high uniformity Si NCs^19^,^20^,^21^,^22^, deviating significantly from the prediction of quantum confinement. Moreover, the PL of the small sized Si NCs exhibits decreased PL quantum yield (QY) and evidently larger nonradiative rate compared to the larger Si NCs, even with identical procedures of synthesis and surface passivation^7^,^12^,^19^. To date, it is still unknown why the blue shift of the S-band should stop at the “magic” wavelength ~590 nm, rather than continues further towards the green/blue^19^,^21^,^22^. To this end we investigate the electron dynamics in Si NCs using steady state and time-resolved techniques. For the first time we elucidate a nanosecond transient yellow PL band (Y-band) emitting around 590 nm that plays a critical role in influencing the electron dynamics and thus dominates the PL shift. When the PL peak approaches the yellow band the excited electrons are dominantly trapped at the surface states and thus the nonradiative component increases significantly. This inhabits any further shift of the PL peak to the blue/green predicted based on quantum confinement only^7^,^10^,^19^,^20^.

## Experimental Results

**Figure 1:**
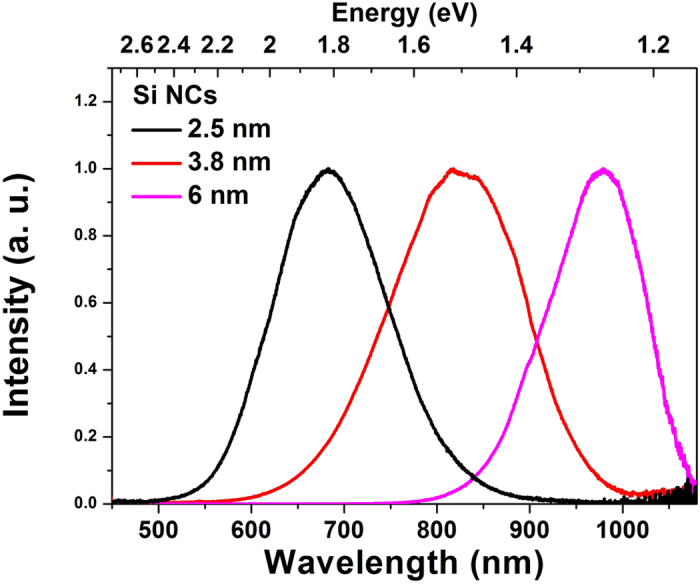
PL spectra of Si NCs of 2.5, 3.8 and 6.2 nm diameter.

Figure 1 shows the steady state PL spectra of 2.5, 3.8 and 6.2 nm Si NCs with excitation at 405 nm. The PL peak exhibits a blue-shift with decreasing NC size due to enhanced quantum confinement, consistent with the other observations^18^,^23^,^24^. It is worth noting that the PL spectra of each sample of Si NCs are essentially invariant as a function of excitation wavelengths between 400 and 500 nm. PL evolution was observed in the μs timescale for 2.5 and 3.8 nm sizes of Si NCs (Figure S4)^25^. The PL evolution typically exhibits a stretched exponential decay and the emission lifetimes evidently decrease with decreasing wavelength^7^,^26^,^27^.

**Figure 2:**
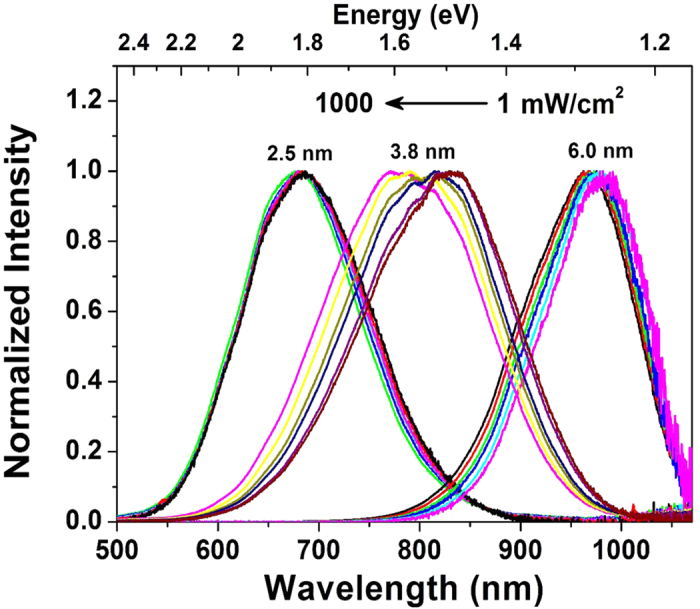
Excitation fluence dependent PL spectra in Si NCs of 2.5, 3.8 and 6.2 nm diameter.

The PL spectra were measured as a function of excitation fluence. A PL peak shift can be observed in each sample with increasing the excitation fluence, which is ascribed to the “state filling” effect^9^,^28^. Upon increasing excitation fluence, the Si NCs of 6.2 nm exhibit a small blue shift, while the 3.8 nm Si NCs exhibit a significantly more marked blue shift, as shown in Fig. 2. It is interesting to note that the 2.5 nm Si NCs do not exhibit the largest blue shift which would be expected on the basis of purely quantum confinement.

**Figure 3:**
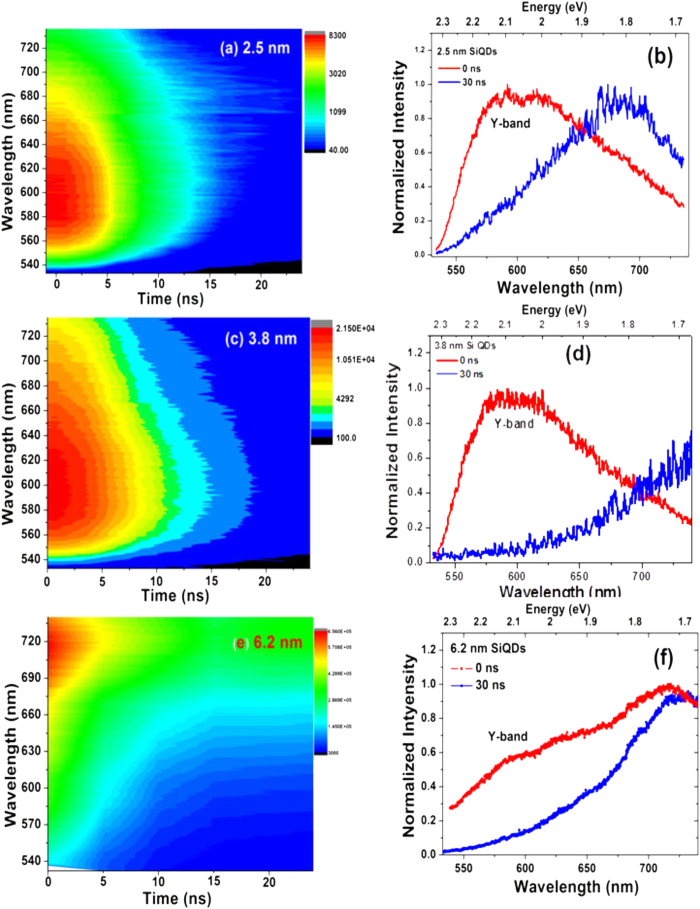
Nanosecond transient PL, (**a**,**c**) and (**e**) PL spectra-time contour; (**b**,**d**) and (**f**) PL spectra at time of zero and 30 ns for Si NCs of 2.5, 3.8 and 6.2 nm diameter.

The time-gated PL is broad, spanning from 540 nm to 800 nm, however, it evolves on the ns timescale from a band centred around 590 nm, hereafter refer as to Y-band, to one significantly further to the red that decays on the μs timescale; as shown as contour plots in Fig. 3a,c,e; and as discrete early and late gated spectra in Fig. 3b,d,f for each sized Si NCs. For the 2.5 and 3.8 nm Si NCs the Y-band is evidently far more significant at early times than the red emission. This is in contrast to the 6.2 nm sample, in which the short wavelength emission is noticeably weaker. The Y-band cannot be observed in the steady state PL spectra (Fig. 1) because the integrated intensity is much lower over this region than the longer-lived emission.

**Figure 4:**
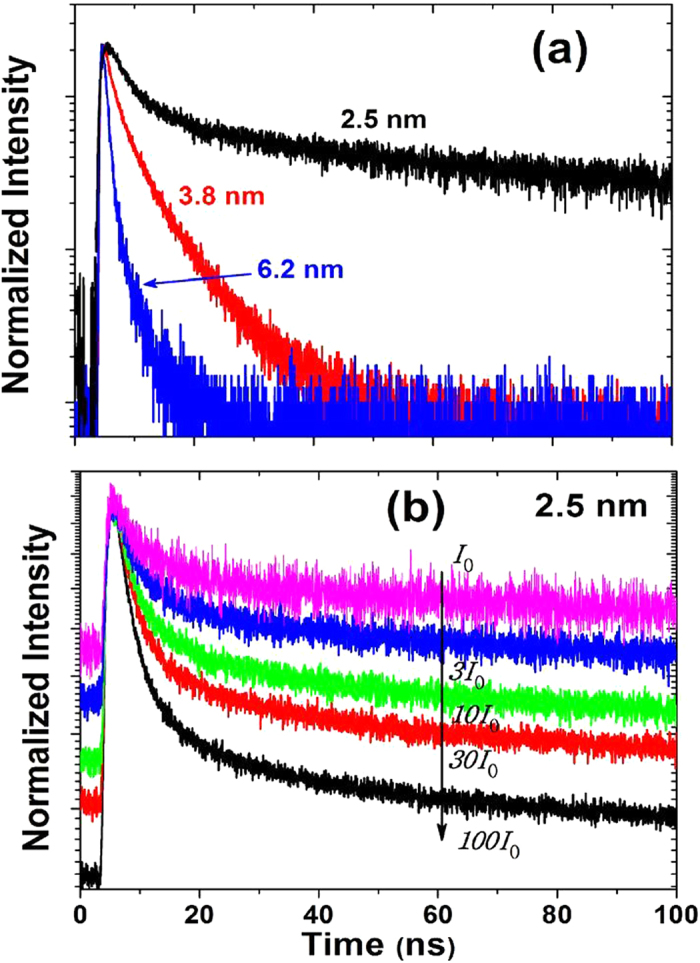
PL temporal decay of Si NCs of (**a**) 2.5, 3.8 and 6.2 nm diameter at λ_m_ = 590 nm; (**b**) 2.5 nm Si NCs with various excitation fluences, I_0_ = 50 mW/cm^2^, at λ_m_ = 590 nm.

The PL evolution of the Y-band was measured using the TCSPC technique for each size of Si NCs, as shown in Fig. 4a. The decay constants were determined using bi-exponential analysis *y* = *A*_1_ exp(−*t*/*τ*_1_) + *A*_2_ exp(−*t*/*τ*_2_), from which average lifetimes were obtained using 〈*τ*〉 = (*A*_1_*τ*_1_ + *A*_2_*τ*_2_)/(*A*_1_ + *A*_2_), as tabulated in Table 1. The ns transition can originate from the relaxation of the excited electrons in the core of Si NCs to the lower states, or from trapping by defect states on the surface. Moreover, the excited electrons on the surface can relax radiatively and nonradiatively, or transfer into the core of the Si NCs. The effect of the phonon bottleneck was proposed as a mechanism for slow relaxation in quantum dot systems although the slow relaxation was rarely observed^29^,^30^. Taking into account the high PL quantum yield, between 30–45% for the current samples, the photoexcited electron in surface trap states should transfer dominantly into the core of the Si NCs. Due to the indirect band gap of Si, the PL from electron-hole recombination exhibits a long lifetime on the μs scale; thus it contributes an apparent intensity offset in the PL evolution on the ns timescale.

To obtain further insight into the influence of the Y-band on electron dynamics, we measured the PL evolution on the timescale at 590 nm with different excitation fluences, as shown in Fig. 4b for the 2.5 nm NCs, with the fitting parameters provided in Table 2. For the 2.5 nm Si NCs the PL lifetime exhibits an increase with increasing the excitation fluence. In contrast, the lifetimes do not exhibit any significant variation for the 3.8 nm (Figure S7b) and 6.2 nm Si NCs upon increasing the excitation fluence.

## Discussion

**Figure 5:**
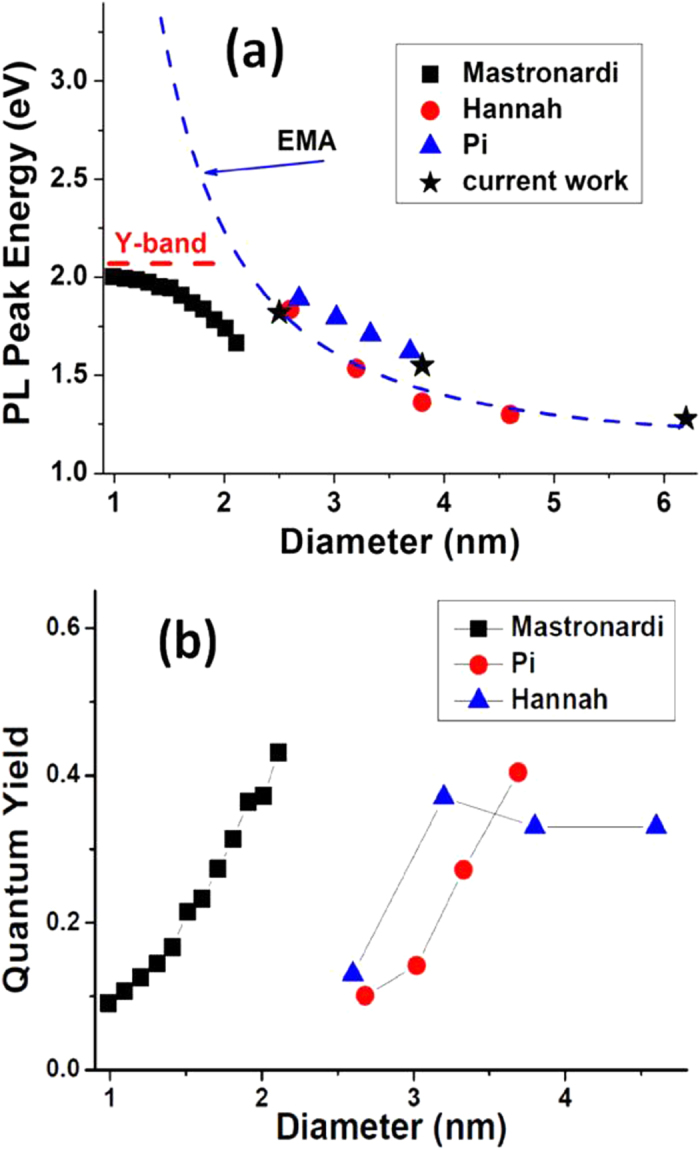
(**a**) Comparison for PL peak energy as a function of diameter of Si NCs from Mastronardi^19^, Hannah^7^, Pi^12^ and current work. The Y-band and the prediction *E*_*g*_(*r*) = 1.12 + 4.57/*r*^2^ are indicated; (**b**) PL quantum yield as a function of diameters of Si NCs from Mastronardi^19^, Hannah^7^, and Pi^12^.

The PL peak energy and quantum yield (QY) as a function of size of Si NCs have been extensively investigated. The size dependence of the PL peak energy is usually predicted theoretically with the effective mass approximation (EMA)^18^: *E*_*g*_(*r*) = *E*_*g*_ + *K*/*r*^2^, where E_g_ is the bandgap of the bulk material and r is the radius of the Si NCs; *K* is a constant. Consistent with many other observations, the PL peak is seen (Fig. 1) to shift significantly towards the blue with decreasing Si NC size, due to enhanced quantum confinement. However, it is rigorously confirmed the PL peak energy significantly deviates from the prediction when the size of Si NCs decreases to 2 nm or smaller; accompanying with a significantly decrease of PL quantum yield^19^,^20^,^21^,^22^. Figure 5 summarizes the PL energy and PL quantum yield as a function of Si NC diameter reported by various groups, compared with the PL peak energy predicted by the EMA. Mastronardi and co-workers found that with NCs of size down to 0.99 nm the peak of PL approaches 600 nm accompanying a reduction in PL QY from 45% to 5% in highly uniform allylbenzene-capped Si NCs prepared using a size-selective precipitation technique^19^. They concluded the significant decrease of QY is due to increased nonradiative trapping processes. Jurbergs *et al.* found that the PL QY of Si NCs decreases when the PL peak wavelength is lower than 700 nm, even arisen from surface processing due to size shrinkage^10^. Hannah and co-workers acquired PL QY of 13, 37, 33, and 33% for 2.6, 3.2, 3.8 and 4.6 nm Si NCs synthesized by the same approach^7^. Pi *et al.* confirmed that the PL peak exhibits a blue shift upon decreasing size but the spectral shift stops around 590 nm with significant reduction in PL QY^12^. Other groups have also reported PL peak blue shifts to around 590 nm with a significant concomitant reduction in PL QY upon decreasing the NC size^7^,^12^,^20^,^21^,^22^.

**Figure 6:**
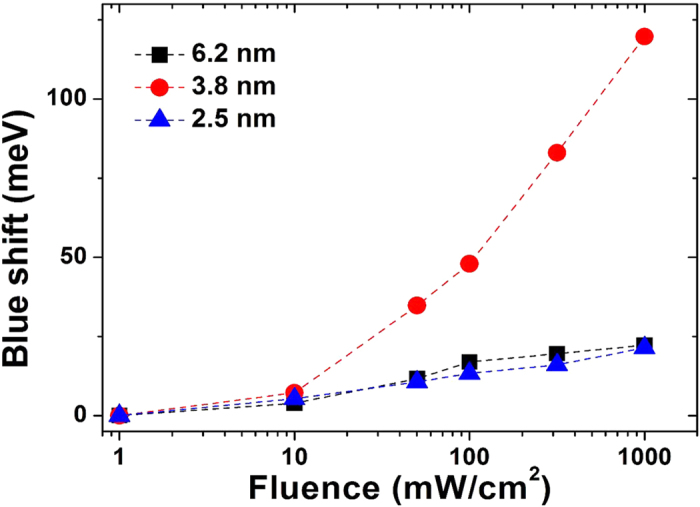
The energy shifts of the PL peak as a function of excitation fluence in Si NCs of 2.5, 3.8 and 6.2 nm diameter.

The observed PL originates predominantly from the lowest excited states in the conduction band at low excitation fluence. The large bandwidth at low fluence is ascribed mainly to size distribution, spectral diffusion and indirect bandgap^20^,^31^. For 405 nm excitation, incident photons can be absorbed by both the Si NCs and the surface ligands, generating electrons in their highly excited states. It is expected that the hot electrons will rapidly cool and then mostly transfer into Si NCs core states. At low excitation fluence the density of photoexcited electrons is low and the excited states are nearly vacant. The hot electrons can transfer very quickly into the core of Si NCs^4^,^32^. Upon increasing the fluence, the excited electrons can fill the higher excited states because the recombination time is much longer than the intraband relaxation time. The blue shift due to the state filling effect depends on the saturation of the lower states of the conduction band. The 6.2 nm Si NCs exhibit little blue shift because of the weak quantum confinement and thus small separation of the discrete states. The Si NCs of 3.8 nm indeed exhibit an apparently larger blue shift than Si NCs of 6.3 nm. However, the PL peak of the 2.5 nm Si NCs exhibits virtually no blue shift upon increasing the fluence, as shown in Fig. 6. Taking solely into account the state filling effect, the smaller Si NCs should exhibit the larger blue shift upon increasing the excitation fluence because of the larger energy separation of the discrete levels. It should be noted that the high energy edge at half maximum exhibits a much smaller blue shift than that of the low energy edge with increasing fluence in the 2.5 nm Si NCs (Figure S5a). As a consequence, the PL band exhibits a small decrease in its apparent bandwidth. In contrast, the high energy edges of the PL band of the Si NCs of 3.8 and 6.3 nm exhibit a far more pronounced blue shift and their bandwidths increase upon increasing excitation fluence (Figure S5b).

Essentially, the electron transfer rate K_*ET*_ can be described on the basis of the Marcus theory^33^,^34^,^35^,


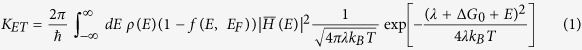


where Δ*G*_0_ = *E*_*CB*_ – *E*_*Y*_ is free energy driving force; 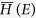 and *ρ*(*E*) are the average electronic coupling and the density of states at energy E relative to the conduction band edge; *λ* is the total reorganization energy. The Fermi occupancy factor, *f*(*E*, *E*_*F*_), ensures that electron transfer occurs only to unfilled states. *k*_*B*_ and T are Boltzmann’s constant and absolute temperature, respectively. The free energy driving force generally dominates the electron transfer rate but the density and the occupancy of the accepting states can also influence the electron transfer rate. For the large sized Si NCs, the larger driving force results from the low energy of the conduction band edge, and the large availability of acceptor states due to small energy separation of the discrete states (Figure S6). The factors result in a large electron transfer rate, consistent with our observation in the larger sized Si NCs.

For 6.2 nm Si NCs, both the intraband relaxation from the high energy excited core states to the lower states as well as electron transfer from the surface relevant states into Si NC cores should be responsible for the observed fast component. The slow component can be attributed to the electron-hole recombination in the Si core. For the large (6.3 nm) Si NCs, it is expected that the PL of the Y-band is very weak owing to effective depletion of the number of excited electrons through electron transfer and intraband relaxation. With decreasing size of the Si NCs the quantum confinement is enhanced. Both the conduction band edge and the energy separation of the discrete states increases, which results in a decrease of the driving force and the availability of the accepting states. A decreased intraband relaxation/electron transfer rate is anticipated and thus the longer decay time is observed.

Upon increasing the excitation fluence, the intraband relaxation rate can be decreased if the occupancy of the excited state increases remarkably. At a critical condition of high excitation fluence and strongly quantum confined Si NCs, in which the higher excited states of Si NCs are filled over the Y-band, it is possible that the excited electrons overflow reversely from the excited states of the Si NCs to the surface states. For the 2.5 nm Si NCs an offset is observed in the PL evolution, due to the spectrally overlapped μs long lifetime emission from e-h recombination in the Si NCs core. This indicates the electrons are partly filled into the higher excited states. It should be emphasized that the relaxation pathway of the photoexcited electrons includes both intraband relaxation in the Si NCs core and to the ligand through interfacial states. In this case, the ns component most likely corresponds to nonradiative trapping to the defect states and radiative recombination on the surface/interface.

It is confirmed the μs component in the 2.5 nm Si NCs is fluence independent (Fig. S7a), because the e-h recombination rate is basically determined by the overlapping of the wavefunction of the electrons and holes. The weight of the fast component (Fig. 4b and Table 2) increases and thus the average decay time decreases with increasing fluence. This indicates the electron transfer is not the major mechanism because increasing occupancy will result in a decrease of the electron transfer rate from surface relevant states to Si NC cores. Instead, the fast and slow components can be ascribed to nonradiative trapping and radiative recombination on the surface, respectively. For such small sized Si NCs, both the occupancy of the higher excited states and the weight of nonradiative trapping increase upon increasing excitation fluence, which suggests the photoexcited electrons in Si NCs cores overflow and then are trapped by defect states on the surface. In contrast, the ns PL evolution was found to be fluence independent in the 3.8 nm Si NCs, (Figure S7b), which implies the density of the excited electrons is not sufficient to affect significantly availability of the accepting states due to the small separation of the discrete levels.

**Figure 7:**
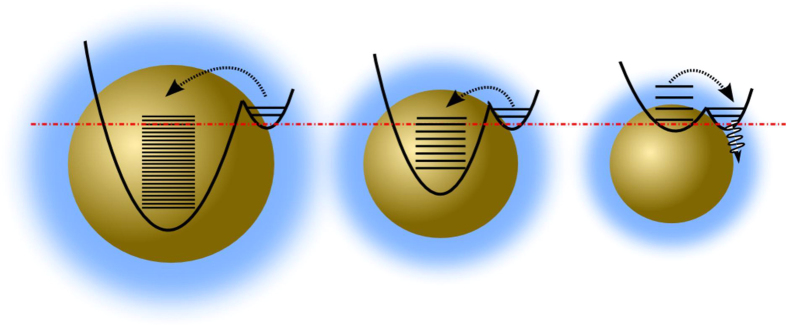
In the large size regime the large driving force and large availability of the accepting states result in a large electron transfer rate.In contrast, a small rate of electron transfer is expected due to decreased energy difference and availability of accepting states. In a critical case that the conduction band edge of Si NCs approaches to the Y-band, the rate of electron transfer to Si NCs significantly decreases and nonradiative trapping becomes dominant.

Wolkin *et al.* proposed a model invoking silanone related mid-gap states in porous silicon, suggesting that there exists a critical size below which quantum confinement increases the separation between the conduction and valence bands and allowing surface localized trap states to influence the PL^36^. Taking into account the influence of the Y-band, when the PL peak of Si NCs approaches the Y-band it is anticipated that (1) the nonradiative trapping of the photoexcited electrons will increase significantly; (2) the PL QY will decreases; and (3) the blue shift of the PL peak decreases or even vanishes with any further decrease the size of the Si NCs. In other words, it is not possible to tune the PL peak (S band) higher than the Y-band (2.1 eV) by decreasing the size of the Si NCs. Due to the presence of the Y-band, the green/blue PL must originate from the surface relevant states, rather than the core of the Si NCs. As shown in the Fig. 7, the relationship between the Y-band and the excited states of the Si NCs can significantly influence the carrier trapping, which will determine the total PL quantum yield. It should be emphasized that the electron transfer rate from the surface into the core is larger for the smaller Si NCs, which results in lower quantum yield due to increased nonradiative defect trapping. The carriers in the surface states have a significantly high possibility to be trapped nonradiatively by defects if they cannot be effectively transferred into the core of the Si NCs.

It should be noted that the Y-band is spectrally not dependent on the size of the Si NCs, which suggests its origin is not the quantum-confined states of Si NCs. The short-lived intravalley direct-band transition can also been ruled out because it has been confirmed the emission energy decreases upon decreasing the size of Si NCs^4^. PL decaying on ns timescale has been observed elsewhere from Si NCs in the blue/green region, and is attributed to O related defect emission^1^,^5^,^37^. We exclude this as the origin of the Y-band emission observed here but rather ascribe it to a surface relevant state, the Si-C bonds. This is based on studies of carefully air-isolated samples, and the surface passivation of the Si NCs induced by hydrosilylation and formation of Si-C bonds. Many kinds of organic molecules, such as derivatives of various alkenes and alkyl groups, can be grafted to the surface of Si NCs, which results in Si-C surface-bond capping^22^,^38^,^39^,^40^,^41^. Hydrosilylation results in hydrocarbon chains being attached to the surface of Si NCs via the Si-C bonds and produces an organic layer with excellent stability against oxidation^42^,^43^. The featured Si-H_x_ peaks in the Fourier transform infrared spectrum (Figure S8) near 2100 cm^−1^ confirms the formation of Si-C bond without the formation of the heavily oxidized O_3_Si-H species. It has been found that the O related component in the blue/green can be observed if the samples are processed in air/oxygen ambient at a high temperature^44^. The O related band was not observed in our samples because O was carefully isolated during sample fabrication.

It is necessary to emphasize that the “magic” wavelength of ~590 nm (2.1 eV) has been observed in various ligand H-terminated Si NCs, e.g., allylbenzene- and alkyl-capped^7^,^12^,^19^,^20^,^21^,^22^,^45^. This indicates the observed Y-band not only exists due to the presence of the ligand used in this study, 1-dodecene, but also the common organically capped Si NCs. Further investigation is nevertheless required for detailed insight into the origin of the Y-band. Although both the reduced PL peak shift and PL QY have been observed by many groups when the PL peak approaches the Y-band, it should be noted an evident inconsistency regarding the critical sizes for the different groups. It has been challenging to obtain accurate sizes of Si NCs by TEM because the relative difference in atomic number between Si and the underlying carbon support layer (z-contrast) is small; and the typical capping ligands for Si NCs have extremely low volatility^8^; which results in up to 20–40% deviation for the sizes of Si NCs from TEM and from X-ray diffraction or small angle X-ray scattering^8^.

In summary, we have demonstrated that a ns transient Y-band can influence critically the carrier dynamics in colloidal Si NCs. The PL peak energy can be predicted based on quantum confinement only in the large size regime (d > ~3 nm); in which quantum confinement dominants the emission and PL QY is independent on the size of the Si NCs. With further reduction of the NC size, the electrons in the higher excited states can be nonradiatively trapped by surface states, because both the edge of the conduction band and the separation of the discrete states increase and thus electrons can fill the higher states. As a consequence, PL peak deviates significantly from the prediction based on quantum confinement; at the same time, the PL quantum yield drops dramatically. This is the so-called magic wavelength around 590 nm. This finding indicates the PL in the red/near infrared preliminarily originates from the quantum confined core states of Si NCs and exhibits wavelength dependent μs lifetime; while the emission in the blue/green region should originate from the surface relevant states with ns lifetime.

## Materials and Methods

The silicon nanocrystals (NCs) used in this study were synthesized using a non-thermal plasma method and surface processed with a 5:1 mixture of mesitylene and 1-dodecene. As-synthesized Si NCs are covalently capped with a ligand (1-dodecene) through a liquid-phase thermal hydrosilylation reaction; details of the plasma synthesis and surface functionalization are given elsewhere^11^,^46^,^47^,^48^. Hydrosilylation results in hydrocarbon chains attached to the surface of Si NCs via Si-C bonds^42^. For spectroscopic measurement the samples were drop cast from toluene or hexane solution on fused silica slides. All the measurement were performed at room temperature.

Particle diameters of 2.5, 3.8 and 6.2 nm with size distributions of 15–20% determined by transmission electron microscopy (TEM) images (supporting information Figure S1) were produced. Each Si NC sample shows very similar featureless absorption (Figure S2a) and PL excitation (PLE) spectra (Figure S2b) monitored at the PL peaks at 700 and 800 nm for the 2.5 and 3.8 nm Si NCs, respectively. It is interesting to note that the very large apparent Stokes shift between the PL and PLE can be due to the very low density of states near the band edge^8^. Steady state PL spectra were recorded using a spectrophotometer comprising a 405 nm laser as the excitation source and a thermoelectrically cooled Si CDD detector. The PL evolution was measured by the time-correlated single photon counting (TCSPC) technique (Microtime200, Picoquant), excited at 405 nm with repetition of 200 kHz. The time resolution of the system is 200 ps. The time-resolved PL spectra were recorded using a gated intensified CCD camera (Princeton Instrument ICCD Max, minimum gate is 2 ns) coupled to a spectrometer (Acton SpectroPro300i) with a 300 l/mm grating. The excitation source was a 355 nm laser-pumped optical parametric oscillator (OPO) with output at 450 nm. The laser pulses have duration of ~7 ns, and a repetition rate of 5 Hz. It has been reported^49^ that when using ICCD systems for gated PL spectra, an “irising” effect may introduce spectral artifacts as a result of the transient gate closing process when the detection gate width is comparable to the opening/closing time and the gate is pretriggered with respect to the signal onset. Figure S3a and 3b show the PL spectra of the 2.5 nm Si NCs recorded with different grating central wavelengths of 600 and 670 nm, respectively, illustrating no significant spectral distortion in the middle of the ICCD chip/central wavelength of grating, confirming that the irising effect is negligible in these measurements in which we applied a 5 ns time window (much larger than the irising time).

## Additional Information

**How to cite this article**: Wen, X. *et al.* Tunability Limit of Photoluminescence in Colloidal Silicon Nanocrystals. *Sci. Rep.* **5**, 12469; doi: 10.1038/srep12469 (2015).

## Supplementary Material

Supplementary Information

## Figures and Tables

**Table 1:** Fitting parameters and average lifetimes at λ_m_ = 590 nm.

Diameter (nm)	2.5	3.8	6.2
τ_1_ (ns)	3.52	1.59	0.83
τ_2_ (ns)	41.2	6.67	4.94
〈*τ*〉 (ns)	14.9	3.53	0.94

**Table 2:** Decay times, weights of the fast component and average lifetimes at various fluence for the 2.5 nm Si NCs at λ_m_ = 590 nm.

Fluence	100 I_0_	30 I_0_	10 I_0_	3 I_0_	I_0_
τ_1_ (ns)	3.18	3.36	3.52	3.95	4.34
τ_2_ (ns)	36.60	38.77	41.21	46.71	55.89
weight of τ_1_ (%)	81	75	70	69	66
〈*τ*〉 (ns)	9.63	12.11	14.97	17.42	21.65

I_0_ = 50 mW/cm^2^.
