# Supplementary material for: Tunability Limit of Photoluminescence in Colloidal Silicon Nanocrystals
Source: Sci Rep. 2015 Jul 22;5:12469. doi: 10.1038/srep12469 (PMC4510486; doi:10.1038/srep12469)
Supplement: Supplementary Information [file srep12469-s1.pdf]

Supplemental information for

Tunability Limit of Photoluminescence in Colloidal Silicon Nanocrystals

Xiaoming Wen,[1] Pengfei Zhang,[1] Trevor A. Smith,[2] Rebecca J. Anthony,[3] Uwe R. Kortshagen,[3] Pyng Yu,[4] Yu Feng, [1] Santosh Shrestha,[1] Gavin Conibeer,[1] and Shujuan Huang[1]*

1: Australian Centre for Advanced Photovoltaics, University of New South Wales, Sydney 2052, Australia
2: School of Chemistry, University of Melbourne, Parkville, VIC, 3010 Australia
3: Department of Mechanical Engineering, University of Minnesota, Minneapolis, Minnesota 55455, United States
4: Research Center for Applied Sciences, Academia Sinica, Taipei, Taiwan

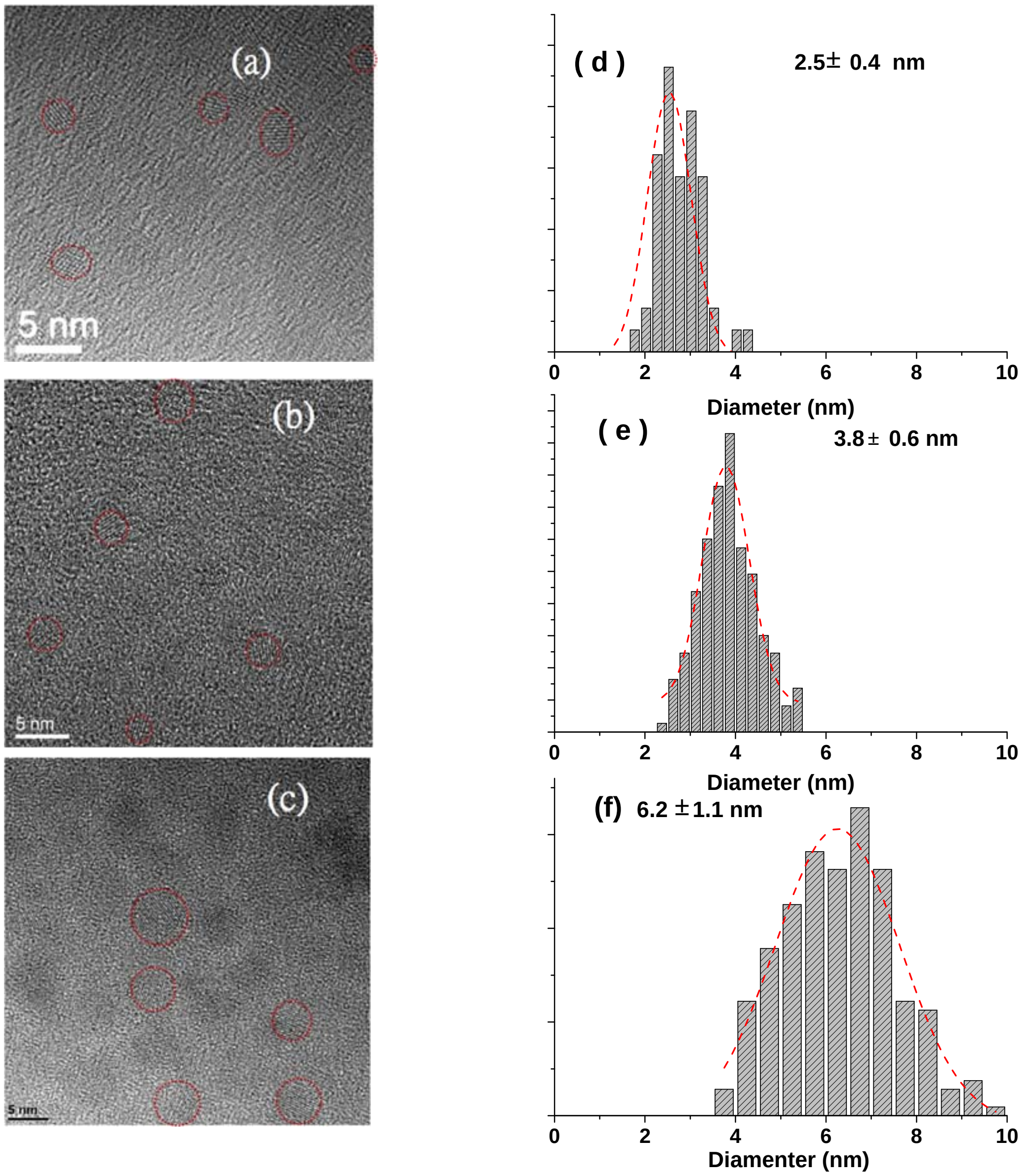

Figure S1 TEM images and size distribution of Si NPs. a-c: HRTEM images of 2.5 nm, 3.8 nm and 6.2 nm Si QDs respectively, lattice fringes are clearly shown; d-f: histograms of corresponded size distributions collected from the QDs images, the dotted red lines are fitted Gaussian profiles, relative sizes of QDs are marked in the form of $d \pm \sigma$ in each graph.

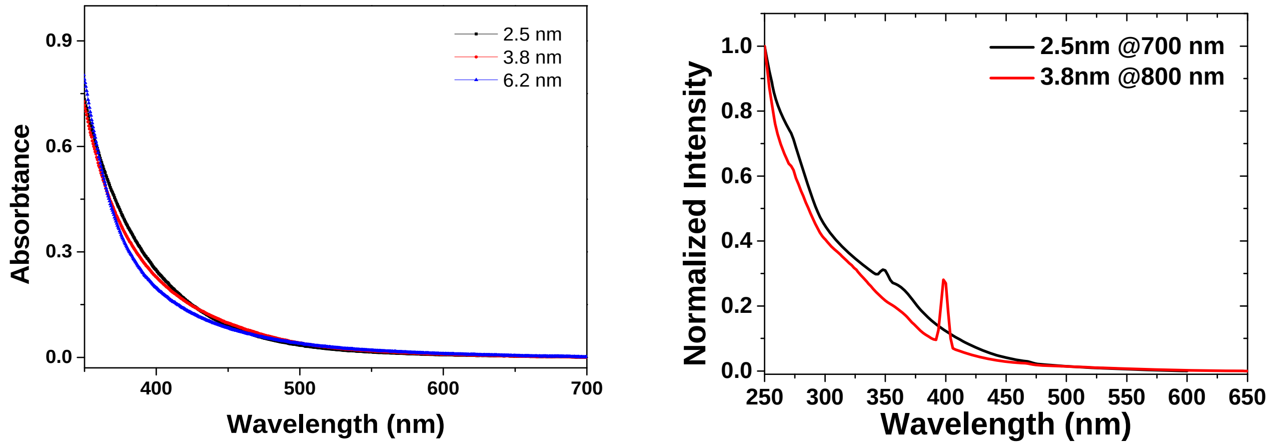

Figure S2 (a) Absorption spectra of Si NCs of 2.5, 3.8 and 6.2 nm. Each Si NCs shows very similar featureless absorption; (b) PLE spectra detected at the PL peak, 700 and 800 nm for 2.5 and 3.8 nm, respectively. Note the peaks at 350 (for 2.5 nm Si NCs) and 400 nm (for 3.8 nm Si NCs) originate from the second diffraction output of the excitation.

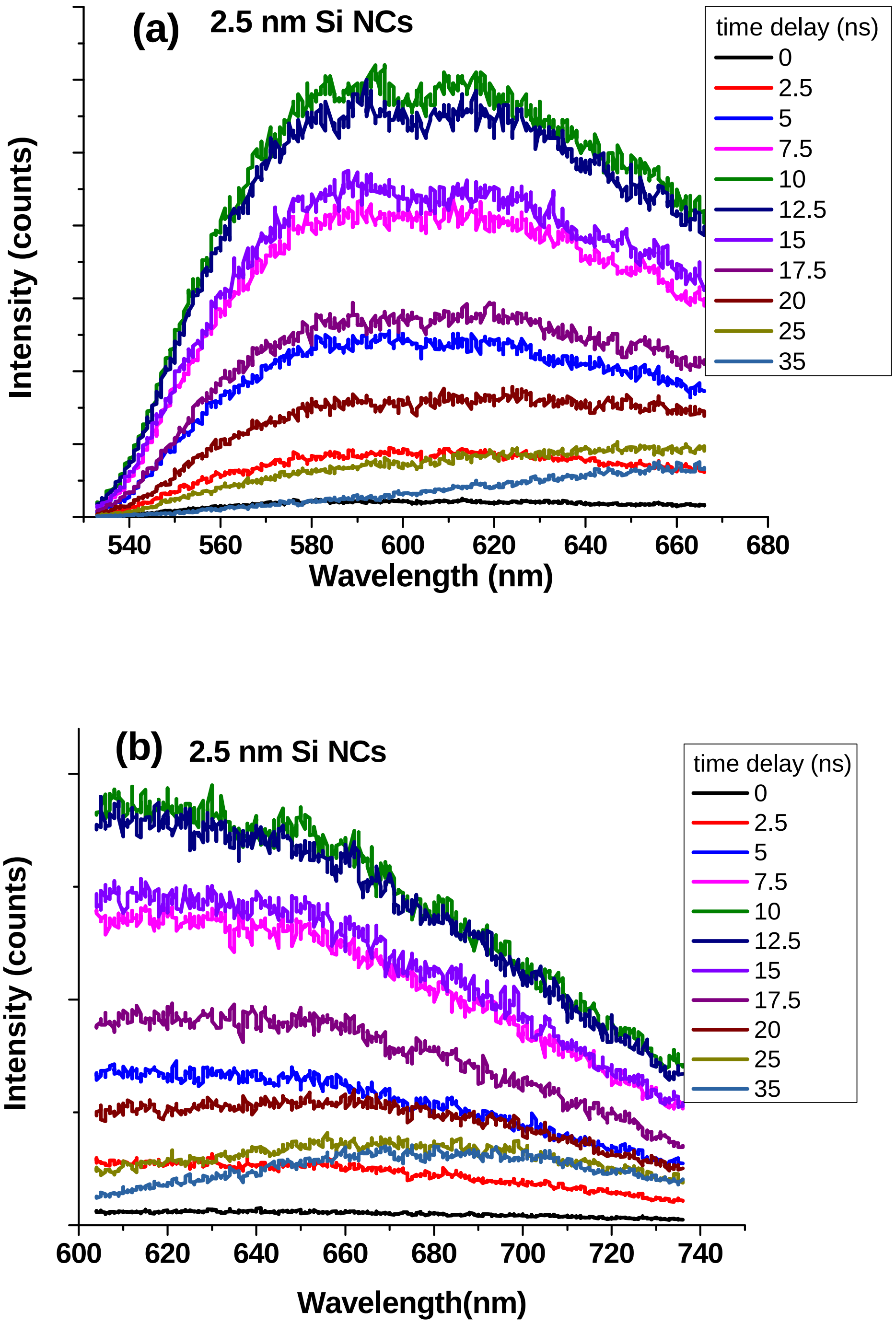

Figure S3 The PL spectra of the 2.5 nm Si NCs recorded with different grating central wavelengths of (a) 600 and (b) 670 nm.

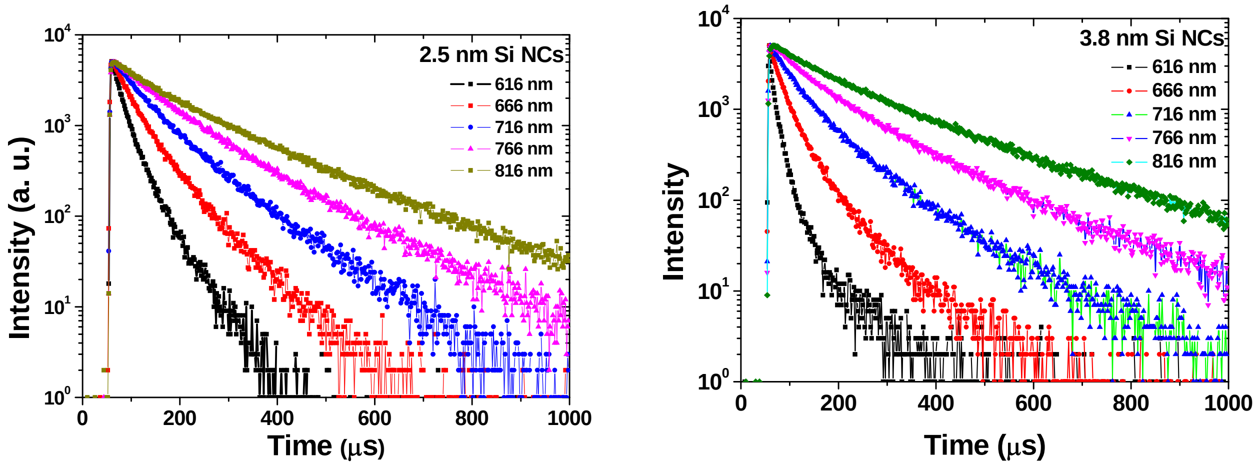

Figure S4. Microsecond PL tracks of 2.5 and 3.8 nm Si NCs.

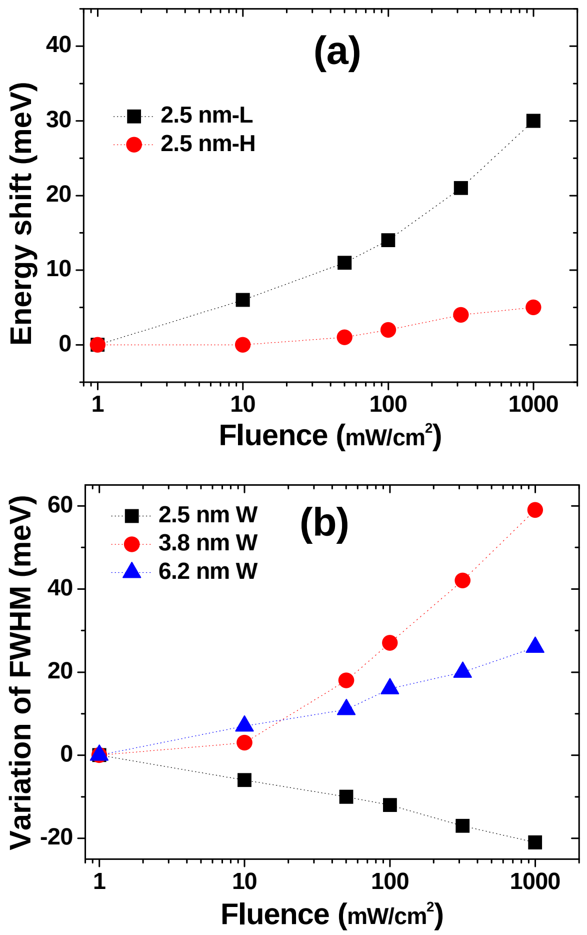

Figure S5. The PL energy shifts as a function of excitation fluence (a) the high and low energy edge at half maximum in 2.5 nm Si NCs; (b) the FWHM of 2.5, 3.8 and 6.2 nm Si NCs.

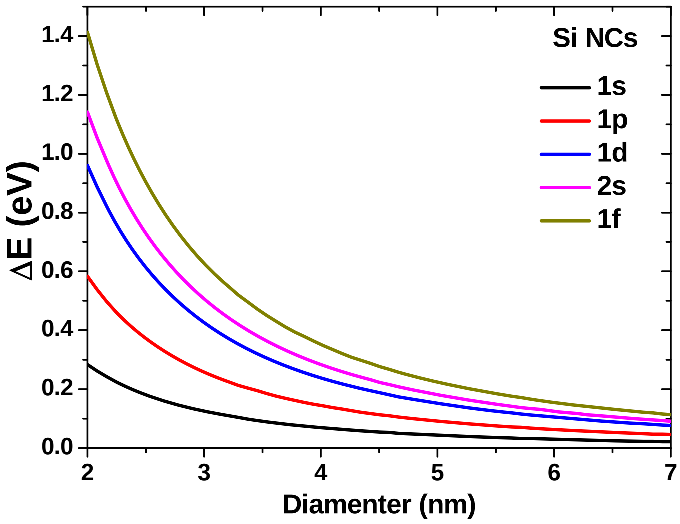

Figure S6. The energy separation of the discrete levels as a function of diameter of Si NCs.

Calculation for the excited states of Si NCs: By using effective mass approximation, the time-independent Schrodinger equation has been solved using standard numerical routines (separation of wavefunction into a radial part and an angular part). A finite potential barrier outside the spherical particle has been considered, yielding energy levels lower than those confined in an infinite barrier. The results show that the lowest three energy levels all correspond to the first eigenstates of their respective radial equations. However they are of different angular momentums (angular quantum number l=0, 1 and 2). The forth level lies at the second eigenstate of the s-orbital radial equation while the fifth level goes back to the first eigenstate but with l=3.

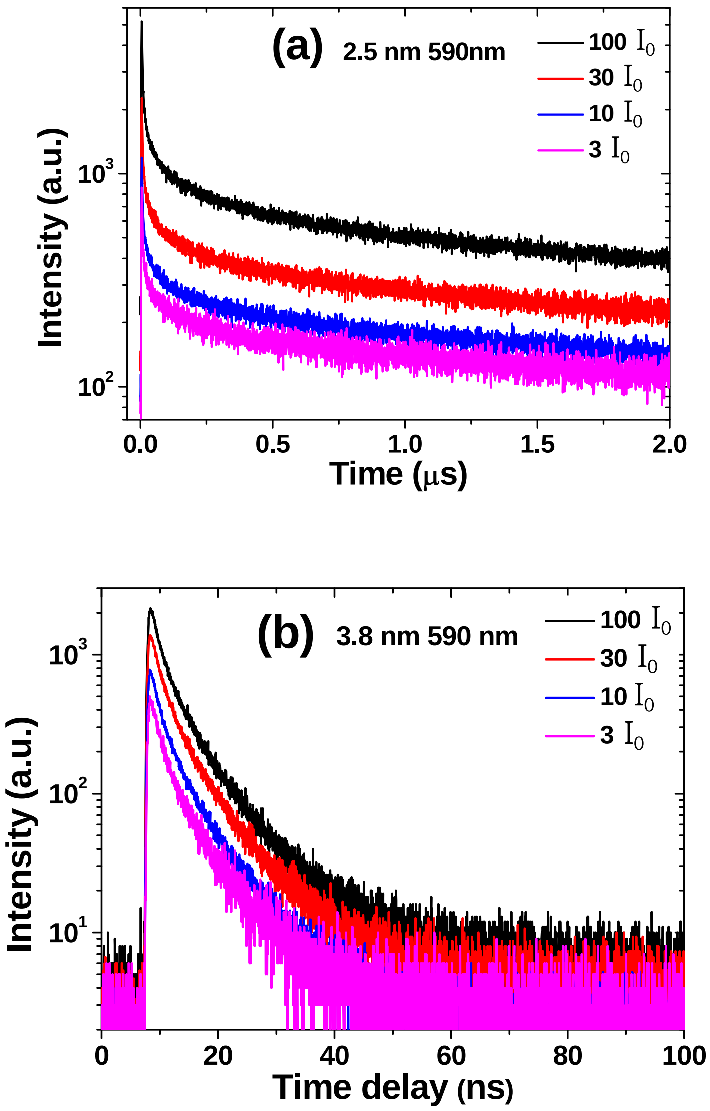

Figure S7. The PL time tracks of Si NCs of (a) 2.5 nm in μs timescale; (b) 3.8 nm in ns timescale at various excitation fluence. $I_0 = 50\,mW / cm^2$

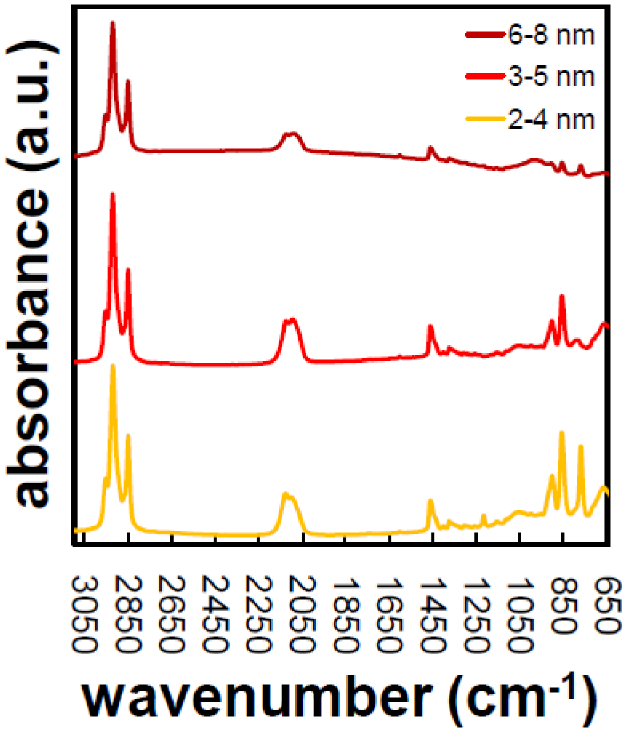

Figure S8. FTIR spectra of Si NCs of 2.5 nm, 3.8nm and 6.2nm; FTIR was measured on drop-cast films of surface-functionalized Si NCs.
